# Obituary: Remembering Professor David Cooper

**DOI:** 10.1186/s12977-018-0418-1

**Published:** 2018-05-24

**Authors:** Stuart Turville, Anthony Kelleher

**Affiliations:** The Kirby Institute, UNSW Australia, Sydney, NSW 2052 Australia


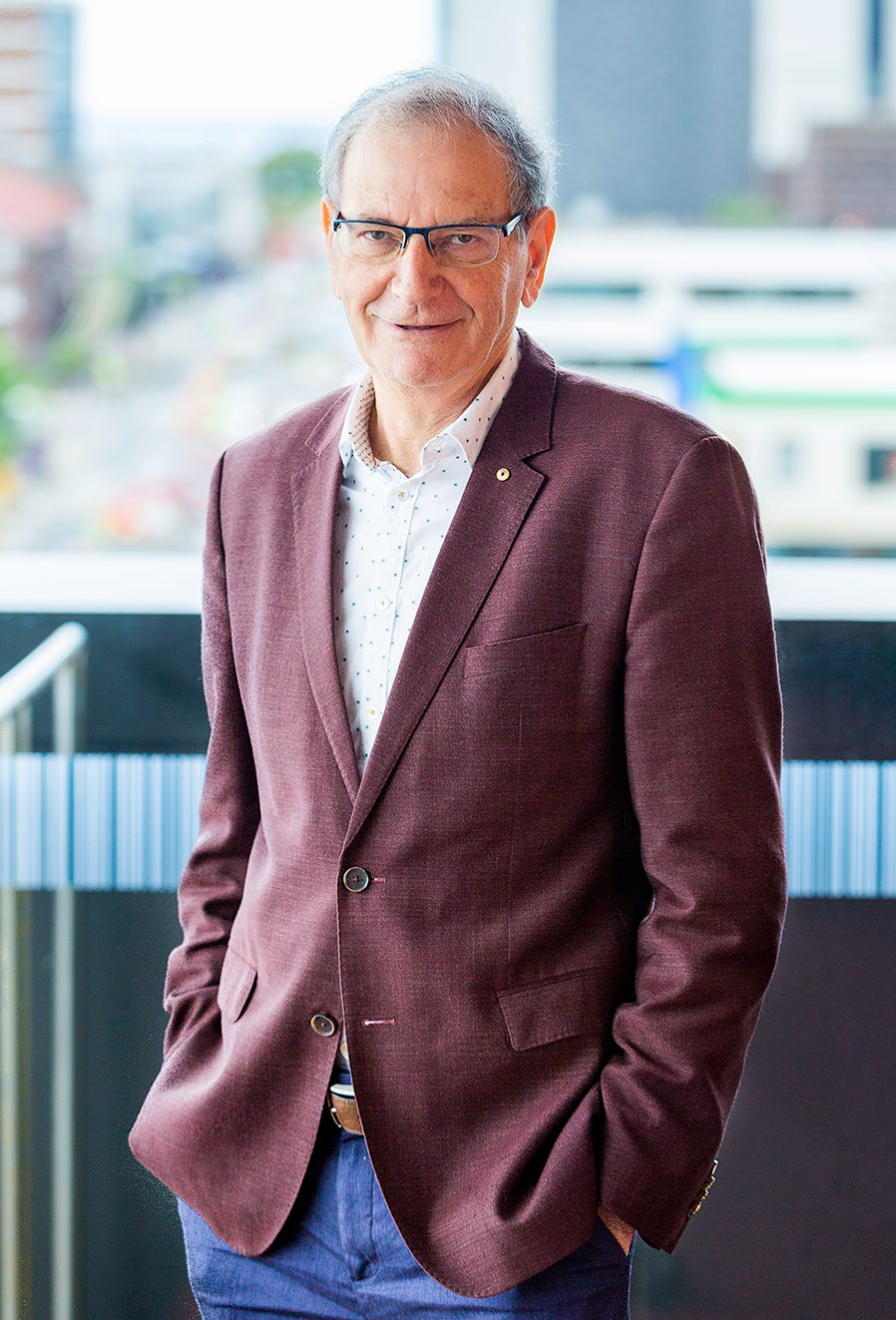
Scientia Professor David Cooper, founding director of the Kirby Institute, died on Sunday 18 March. His work on HIV infection made him an internationally renowned clinician scientist and advocate.

The name David Cooper has, over the past three decades, become synonymous with HIV research and treatment in Australia. Throughout his career, he was consistently at the forefront of clinical and immunological research into the virus; his inquisitiveness coupled with his compassion for his fellow person were traits that he possessed from a very young age. Born in Sydney in 1949, Cooper finished high school aged 15 and immediately commenced medical studies at the University of Sydney against advice to have a year or two off. He graduated as a doctor in 1972, with first class honours and a couple of research publications. By 1980 he had obtained fellowships in internal medicine and pathology, qualifying him as an immunologist, and had completed his Doctoral degree in human B cell biology.

It was during his post-doctoral stint at the Dana Farber Cancer Institute in Boston in 1981 that Cooper first learned of an unknown and aggressive immune deficiency syndrome that was highly prevalent in gay men and intravenous drug users (IDU). Having seen the devastating early effects of AIDS on the immune system in the US, Cooper returned to the Department of Clinical Immunology at St Vincent’s Hospital, Sydney in 1983. He anticipated not only that a similar epidemic would reach Australia, but that it would affect those same populations, meaning that St Vincent’s, being located close to the hub of gay culture in Sydney, would become an epicentre of the HIV epidemic in Australia. His hypothesis was proven correct, with HIV spreading rapidly among 4500 people, predominantly gay men, from 1983 to 1985.

Cooper really set the foundation for Australia’s response to the HIV epidemic in these early days, with the establishment of one of Australia’s first HIV cohort studies in 1983. Cooper and colleagues swiftly enlisted over 1000 gay men into a prospective study that collected clinical data and biobanked samples to begin searching for answers in anticipation of a frightening epidemic. The results of this clinico-pathological study were seminal in Australia’s, and indeed the world’s, response to HIV, with Cooper explaining for the first time the events of “seroconversion illness”, or primary HIV infection, in his 1985 paper published in *The Lancet.* This collaborative study, centred on patients and linking primary and tertiary clinical care with state of the art laboratory studies and epidemiological expertise, set the basic strategy for Cooper’s work for the next 30 years. These original studies provided insights into pathogenesis, as well as providing the first detailed understanding of the epidemic’s reach and impact. In 1986, he founded the National Centre in HIV Epidemiology and Clinical Research, which later became the Kirby Institute.

At the same time, despite the pervasive negativity driven by fear and stigma, Cooper set up a comprehensive clinical service including Australia’s first HIV/AIDS ward at St Vincent’s, supported in full by the Sisters of Charity, who ran the hospital at the time. He gathered a team of physicians, nurses, allied health professionals and laboratory scientists with complementary expertise to provide excellence in clinical care, setting the benchmark for clinical services responding to the crisis that HIV brought to the local community. “It was completely devastating, and we just couldn’t keep up”, said Cooper in a 2015 interview with the ABC.

Bringing together the clinical and research aspects of his ambition, he used his collaborative networks as a vehicle for clinical trial research, ensuring Australian patients had early access to all new therapies whilst robustly assessing the efficacy and toxicity of each new intervention.

Not only was Cooper a leader in Australia’s response to HIV, he was also an influential figure in the global response, particularly in low and middle-income countries. His leadership as President of the International AIDS Society from 1994 to 1998 was instrumental in highlighting the lack of treatment in resource poor areas hit hard by the HIV epidemic. Cooper had a particular focus on South East Asia, being a logical sphere of influence for Australia.

One of his most prominent successes is HIV-NAT, a research partnership between the Netherlands, Australia and Thailand. Established in 1996, HIV-NAT is dedicated to capacity building and improving access to HIV treatments in Thailand and beyond. As one of the three founding directors, Cooper instigated many of its activities. HIV-NAT has brought world-class research capacity to South East Asia and is now a fully independent Thai-led organisation recognised as the regional leader in all aspects of HIV research.

In Australia, he remained at the forefront of HIV clinical research. With his colleagues at St Vincent’s, he provided the earliest descriptions and pathological underpinnings of key side-effects of the new treatments, including the lipodystrophy syndrome, which inspired new directions in drug development. He took a leading role in the design of many of the most important international trials that have led to the optimal use of simplified, more effective and less toxic regimens. The results of these trials have determined current treatment guidelines and have played a large role in the extraordinary advances in the treatment of this once universally deadly disease.

Whilst Cooper readily succeeded as a brilliant clinical researcher, his former and current patients always spoke of a doctor who would provide reassurance in the face of what was universally a frightening and, at the time, little understood illness. He always made himself available, whether by phone or email, and particularly in the early days, by the hospital bed.

Through his clinical leadership and compassion, Cooper was significant contributor to what is now referred to as the “Australian model”, exemplified by our swift and egalitarian response to HIV. Key to the success of preventing HIV transmission has always been the willingness of highly marginalised risk groups to have a dialogue with doctors and politicians in order to better understand how the virus spreads and how best to stop it spreading further. Cooper was a major facilitator of collaboration in the early days, despite others tension and hesitation, and his inclusive approach to health was a guiding principle throughout his career.

Cooper’s focus was on the clinical face of HIV, however his leadership inspired a spectrum of HIV researchers across a range of disciplines. He readily understood the power of all aspects of research into this disease. In basic science, he brought a level of pragmatism that grounded our work. For instance, whilst readily supporting our basic science endeavours, he would remind us of the practicalities of implementation. Often, he would ask how a particular study might directly impact the lives of the patients he was caring for.

For the younger scientists he also shared with us the memories of a time when the epidemic hit hard and lives were being lost at a rapid rate. He lost many friends and colleagues during this time and stressed that whilst significant progress had been made over the past three decades, it has come at a great cost.

Those with HIV who have access to diagnosis and treatment can now live healthy and long lives; with undetectable viral loads, the probability of transmission is also dampened. Further effective HIV drugs can provide greater choice for prevention in those who are not HIV positive with now-widespread access to PreP. One of David’s last studies was a demonstration project of rapid roll-out of PreP in high risk individuals with the ambitious goal of reducing incidence at a population level. David never failed to remind us that this level of testing and treatment was not always the case in the early epidemic. “We lost many beautiful people during this time”, he often said. Whilst he is sadly no longer with us, these words still inspire.

Scientifically, our last conversations with David as basic scientists centred on developing a cure for HIV. Whilst we have had great success in HIV therapy, the obvious next step must be to enable a life for people living with HIV in the future that was free of a daily antiretroviral treatment regimen. Whilst this remains a challenge, David firmly set the framework for this reality to be realised, and it is the practice of translational research, which David firmly advocated, that will enable equal access to health in the future.

Tributes

*Michael Kirby: Former Justice to the High Court of Australia*

“David’s special gift was having both a huge intellect and a huge heart. It was his intellect that made him a leader in the global response to the AIDS epidemic and led to the building of the Kirby Institute. But it was his great heart that all who knew him, his family, his colleagues and his patients, could witness every day. He was first a clinician, and that made him a great scientist. We will miss him terribly and be all too aware of his absence.”

*Professor Anthony Kelleher: Program Head Kirby Institute and Acting Dean University of New South Wales, Australia*

“David’s importance as a clinician scientist in the field of infectious diseases cannot be overstated. He contributed to the development of every therapeutic drug used in HIV. All over the world he was respected as a leader, and at home he was an insightful colleague and unparalleled mentor.”

*Professor John Kaldor: Program Head Kirby Institute, University of New South Wales, Australia*

“His research had a direct and life-saving impact for so many people, in Australia and all over the world. Our Institute is David’s legacy, and while we are devastated to lose him, we carry on his work with immense dedication, honour and pride.”

*Professor Basil Donovan: Program Head Kirby Institute, University of New South Wales, Australia.*

“A giant in his field with a huge heart”.

*Professor Phanuphak Praphan, Director of the Thai Red Cross AIDS Research Centre*

“Let those of us who remain become stronger to follow David’s footsteps and put an end to AIDS.”

*Professor Brendan Crabb, CEO of the Burnet Institute, Melbourne Australia*

“So many once highly vulnerable people are alive, live healthily and are empowered because of David. While he leaves an incredible and enduring legacy, his loss is immense”.

*Dr Justin Koonin, AIDS Council of New South Wales (ACON), Sydney Australia*

“David’s contribution to the health and wellbeing of people affected by HIV and LGBTI people has been immeasurable. We have all benefitted from his uncompromising principles and integrity, his passion, his fierce intelligence and intellect, his pioneering spirit and his compassion.”

*Owen Ryan, International AIDS Society (IAS) Executive Director*

“Throughout his career, David continued to consult as a physician and was renowned for his compassion with each of his patients. That perfectly encapsulates the genuine heartfelt nature of who David was and how he approached his work. We are forever indebted to him for his vision, tenacity and humanity.”

*Professor Linda*-*Gail Bekker, IAS President*

“David played a crucial role in developing the drug trials for many of the HIV medications currently on the market and saving lives today. He was a brave researcher, an invaluable collaborator and is an irreplaceable force in the HIV community.”

